# Comparative analysis of RNA expression identifies effective targeted drug in myoepithelial carcinoma

**DOI:** 10.1038/s41698-025-00918-5

**Published:** 2025-05-17

**Authors:** Yvonne A. Vasquez, Lauren Sanders, Holly C. Beale, A. Geoffrey Lyle, Ellen T. Kephart, Katrina Learned, Jennifer Peralez, Amy Li, Min Huang, Kimberly A. Pyke-Grimm, Serena Y. Tan, Sofie R. Salama, David Haussler, Isabel Bjork, Olena M. Vaske, Sheri L. Spunt

**Affiliations:** 1 Department of Molecular, Cell and Developmental Biology, University of California, Santa Cruz, CA USA; 2 UC Santa Cruz Genomics Institute, Santa Cruz, CA USA; 3 Department of Biomolecular Engineering, School of Engineering, University of California, Santa Cruz, CA USA; 4 Stanford University School of Medicine, Stanford, CA USA; 5 Department of Nursing Research and Evidence-Based Practice, Stanford Medicine Children’s Health, Stanford, CA USA; 6 Present Address: Blue Marble Space Institute of Science, NASA Ames GeneLab, Silicon Valley, CA USA; 7 Present Address: Foundation to Advance Vascular Cures, Redwood City, CA USA

**Keywords:** Cancer, Computational biology and bioinformatics

## Abstract

Myoepithelial carcinoma is an ultra-rare pediatric solid tumor with no targeted treatments. Clinical implementation of tumor RNA sequencing (RNA-Seq) for identifying therapeutic targets is underexplored in pediatric cancer. We previously published the Comparative Analysis of RNA Expression (CARE), a framework for incorporating RNA-Seq-derived gene expression into the clinic for difficult-to-treat pediatric cancers. Here, we discuss a 4-year-old male diagnosed with myoepithelial carcinoma who was treated at Stanford Medicine Children’s Health. A metastatic lung nodule from the patient underwent standard-of-care tumor DNA profiling and CARE analysis, wherein the patient’s tumor RNA-Seq profile was compared to over 11,000 uniformly analyzed tumor profiles from public data repositories. DNA profiling yielded no actionable mutations. CARE identified overexpression biomarkers and nominated a treatment that produced a durable clinical response. These findings underscore the utility of data sharing and concurrent analysis of large genomic datasets for clinical benefit, particularly for rare cancers with unknown biological drivers.

## Introduction

Although childhood cancer outcomes have improved over the past few decades, those with high-risk or recurrent/refractory disease still fare poorly^1,2^. The small number of affected patients and disease biologic heterogeneity have led to precision medicine approaches for identifying targeted therapy options for individual patients^3,4^. Tumor genomic profiling has identified clinically useful therapeutic targets in both adult and pediatric cancers^5–8^. Pediatric cancers have fewer somatic coding mutations than adult cancers but may have other genetic alterations such as copy number alterations, gene fusions, and epigenetic mechanisms^9,10^. Adding tumor RNA sequencing (RNA-Seq) to DNA mutation analysis may identify additional treatment options for pediatric cancer patients, but the utility of RNA-Seq has been largely limited to identifying gene fusions^11–17^. A recent study demonstrated the potential value of utilizing tumor RNA-Seq for the diagnostic classification of pediatric cancers^18^. Complicating gene expression analyses, there are no published standards for identifying gene expression outliers, and the impact of using different comparator cohorts for outlier detection has not been studied. Research to date has provided promising evidence that RNA outlier expression analysis can identify clinically relevant targets in the absence of a detectable characteristic genomic alteration (mutation, fusion, etc) through case studies^19–21^ and cohort studies^11,12,22,23^.

To overcome those limitations, our Comparative Analysis of RNA Expression (CARE) approach^24^ compares gene expression in a focus patient sample to those in personalized comparator cohorts derived from large datasets of tumors from multiple institutions and studies. CARE uses a standardized analytic workflow with stringent quality criteria, comparisons to multiple personalized cohorts, and pathway analysis to identify overexpression outliers that are most relevant to pediatric cancers. The outlier genes detected by CARE reflect activation of oncogenic pathways and are used to identify potentially valuable therapeutic targets. This method, including the impact of cohort selection on outlier detection, is described in a companion manuscript^25^. To illustrate the value of the CARE approach, we describe a young child with metastatic recurrence of an ultra-rare cancer, myoepithelial carcinoma, for which there were no known effective therapy options and no actionable mutations on tumor DNA profiling. Following CARE analysis, two targeted therapies not previously reported to be useful in myoepithelial carcinoma were identified and administered. Progressive disease was noted after the first therapy, but the second therapy produced prolonged stable disease. The child remains free of disease after completing two years of this therapy along with resection of residual disease. This case illustrates a framework for analyzing tumors using the CARE approach and integrating this information into clinical decision-making. It also demonstrates why using large comparator datasets and an unbiased analytic approach may help identify potentially useful therapies for patients with rare and difficult-to-treat cancers.

## Results

### Case description and initial presentation

**Fig. 1:**
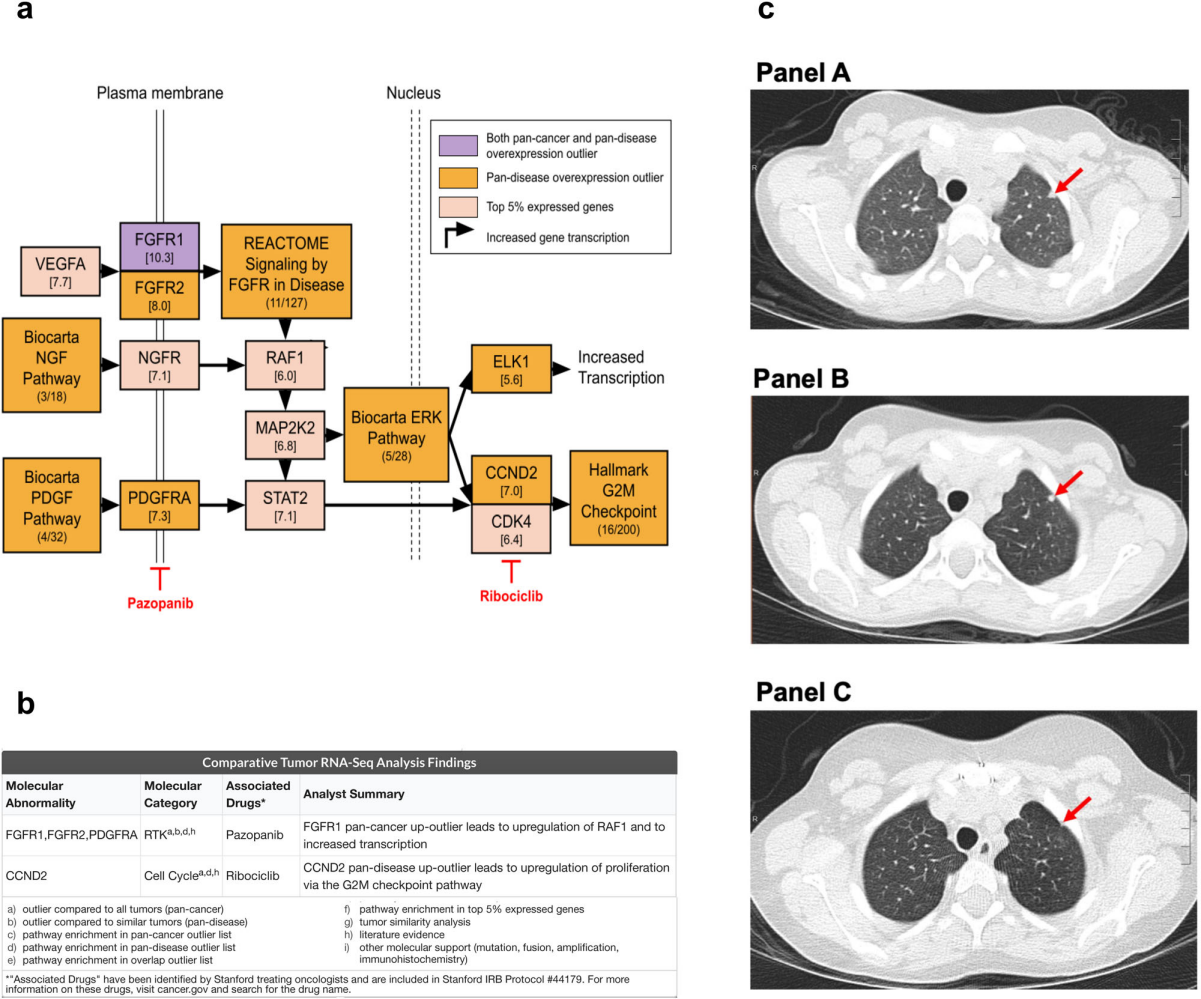
Summary of CARE analysis results and treatment outcomes. **a** CARE pathway enrichment summary. Significantly overexpressed genes are colored according to the legend. Pathway enrichment was assessed by the Molecular Signatures Database. Expression in log_2_(TPM + 1) for individual genes is reported in square brackets. Multiple receptor tyrosine kinases (*FGFR1*, *FGFR2*, *PDGFRA*) were found to be overexpression outliers, consistent with pan-disease enrichment in the “Reactome Signaling by FGFR In Disease” pathway and the “Biocarta PDGF pathway”. *CCND2* was a pan-disease expression outlier and the “Hallmark G2M Checkpoint” pathway was also enriched in pan-disease expression outliers, resulting in upregulation of proliferation via increased cell cycle activity. **b** Summary of clinically actionable findings identified by CARE analysis. Criteria for defining the analytical strength of findings are listed a-i. **c** Axial computed tomography (CT) images without intravenous (IV) contrast enhancement demonstrate a left upper lobe non-calcified pulmonary nodule immediately prior to initiation of ribociclib therapy (Panel A, red arrow) that was stable in size after twelve 28-day cycles of ribociclib therapy (Panel B, red arrow). This lung nodule was subsequently adequately surgically excised and confirmed pathologically to represent metastatic myoepithelial carcinoma. Follow-up axial CT image without IV contrast enhancement shows a stable scar at the site of the resected nodule 31 months after surgery and 18 months after completion of twelve 28-day cycles of postoperative ribociclib (Panel C, red arrow).

The patient presented at 23 months of age with a 12.6 cm hepatic mass. Incisional biopsy showed an INI-1-deficient undifferentiated malignant neoplasm. After two cycles of vincristine/doxorubicin/cyclophosphamide chemotherapy, the tumor was completely excised, and the diagnosis was revised to myoepithelial carcinoma after consultation with the pathologist who defined the clinicopathologic features of this tumor in childhood^26^. Ifosfamide/doxorubicin chemotherapy (cumulative 54 g/m^2^ ifosfamide, 345 mg/m^2^ doxorubicin) was administered postoperatively. The patient remained disease-free until routine surveillance computed tomography (CT) imaging showed three new pulmonary nodules 26 months after therapy completion, one of which was confirmed pathologically as myoepithelial carcinoma (Fig. 1c, Panel A). DNA profiling of one of the metastatic lung nodules identified INI-1 deficiency (*SMARCB1* deletion).

### Patient’s CARE analysis, treatment, and outcome

A metastatic lung nodule was sequenced and the tumor RNA-Seq dataset was sent to UCSC Treehouse for Comparative Analysis of RNA Expression (CARE) to identify targetable overexpression genes and pathways for treatment.

**Fig. 2:**
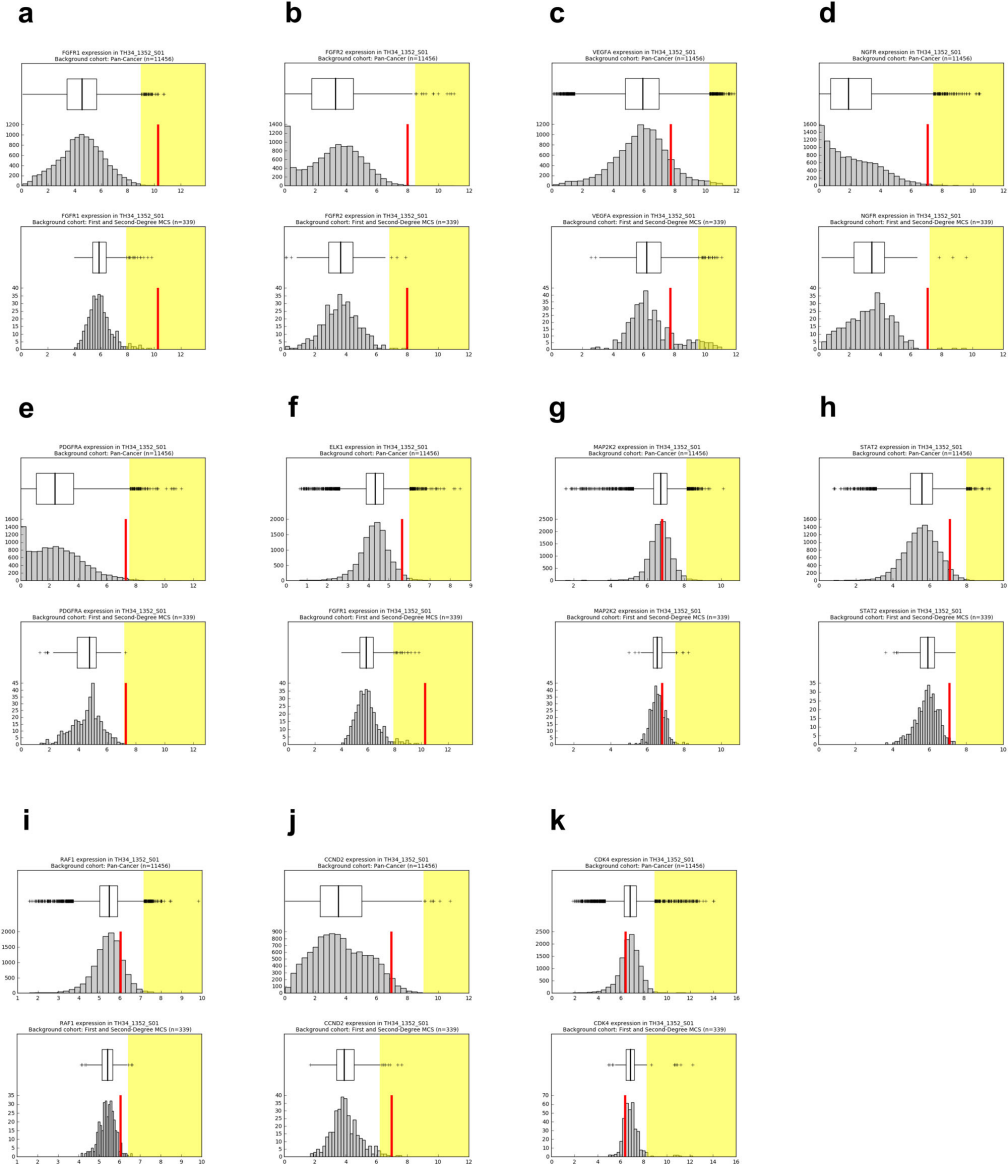
Gene expression levels in the patient’s RNA-Seq dataset relative to comparator cohorts. The expression of each gene of interest (outlier or implicated by pathway analysis) in the patient’s RNA-Seq dataset (TH34_1352_S01) is denoted with a vertical red line plotted with respect to the gene's expression in log2(TPM+1) across the comparator cohort (x-axis). The outlier range is denoted with a yellow bar. a-k. Expression in the pan-cancer (top) and pan-disease (bottom) cohorts for (a) FGFR1, (b) FGFR2, (c) VEGFA, (d) NGFR, (e) PDGFRA, (f) ELK1, (g) MAP2K2, (h) STAT2, (i) RAF1, (j) CCND2, (k) CDK4.

**Fig. 3:**
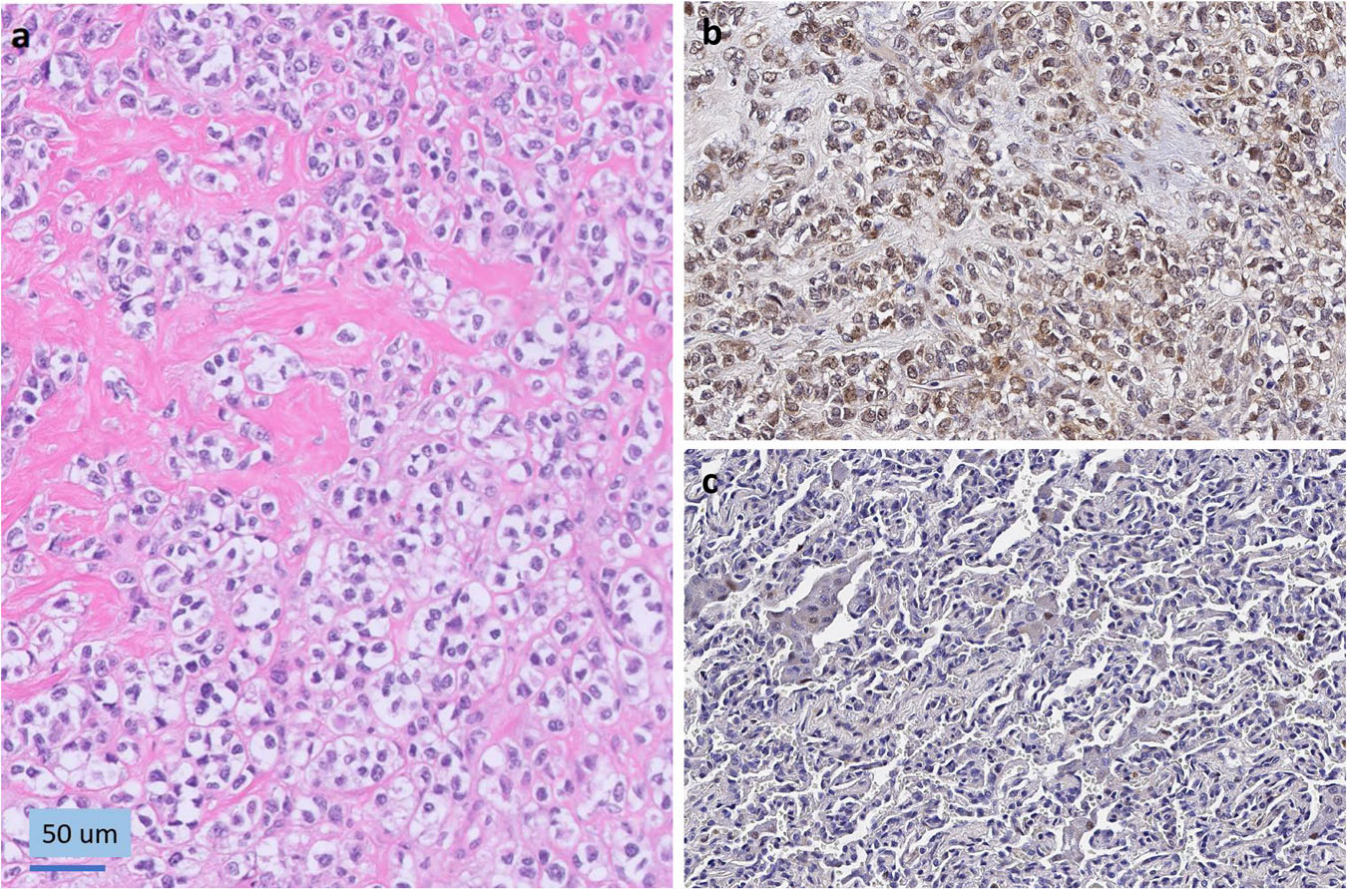
Immunohistochemistry stains for CDK4 expression. **a** Histologic sections of metastatic tumor in the lung demonstrate cords and nests of epithelioid cells with clear cytoplasm. An immunohistochemical stain for CDK4 shows brown staining indicating overexpression in tumor cells (**b**), compared to non-neoplastic lung parenchyma (**c**). **a** Hematoxylin and eosin stain, 20x magnification; **b**, **c**, immunoperoxidase stain with hematoxylin counterstain, 20x magnification).

Both “pan-cancer” and “pan-disease” CARE analyses were performed. For pan-cancer analysis the patient’s tumor RNA-Seq profile was compared to a cohort of 11,427 tumor RNA-Seq profiles from both adult and pediatric patients. Pan-cancer outliers are those exceeding the outlier threshold defined by the entire cohort. In contrast, pan-disease analysis typically compares a patient’s RNA-Seq profile to four personalized pan-disease cohorts: (1) datasets from tumors with the same diagnosis as the patient’s profile, (2) molecularly similar RNA-Seq datasets (first degree neighbors), (3) first and second degree neighbors (first degree neighbors plus RNA-Seq datasets molecularly similar to them), and (4) datasets from diseases present among the top six datasets most molecularly similar to the patient’s profile. Molecular similarity is assessed by Spearman correlation. However, the pan-disease analysis for this case study was limited by the fact that there were only three tumors in the compendium whose RNA expression profiles met the threshold for molecular similarity to our patient’s current tumor expression profile, and these three were also the only myoepithelial carcinomas in the compendium. Those three myoepithelial carcinoma datasets all originated from the case study patient, but were nevertheless useful in identifying additional similar datasets. Thus, the pan-disease analysis for this patient included a single cohort of RNA-Seq profiles from only 339 tumors (Table 1), which consisted of the three myoepithelial carcinoma samples from our patient and 336 tumors that were significantly correlated with them (Spearman correlation above the 95th percentile). Despite this limitation, CARE analysis findings included multiple overexpressed receptor tyrosine kinases (*FGFR1, FGFR2, PDGFRA*) consistent with enrichment in FGFR and PDGF pathway signaling, which is targetable with pazopanib, as well as *CCND2* overexpression with pathway support, which is targetable by ribociclib (Figs. 1a and 2). The research report identified both pazopanib and ribociclib as clinically actionable (Fig. 1b); pazopanib was prioritized based on its efficacy in soft tissue sarcomas and documented safety in children^27,28^ while safety testing of the liquid formulation of ribociclib for young children had not yet been completed^17,29^. A *CDK4* immunohistochemical stain on a metastatic lung tissue sample confirmed *CDK4* overexpression in the tumor but not in the surrounding non-neoplastic lung parenchyma (Fig. 3, Panels B and C); *Retinoblastoma* protein was intact (data not shown).

**Table 1:** Samples used in pan-disease analysis

disease	*n*
breast invasive carcinoma	68
pancreatic adenocarcinoma	67
kidney clear cell carcinoma	46
osteosarcoma	20
lung adenocarcinoma	19
stomach adenocarcinoma	14
colon adenocarcinoma	11
mesothelioma	10
testicular germ cell tumor	9
lung squamous cell carcinoma	8
ovarian serous cystadenocarcinoma	8
thyroid carcinoma	8
bladder urothelial carcinoma	7
dedifferentiated liposarcoma	5
skin cutaneous melanoma	5
glioblastoma multiforme	4
rectum adenocarcinoma	4
cholangiocarcinoma	3
kidney papillary cell carcinoma	3
sarcoma	3
glioma	2
hepatoblastoma	2
myoepithelial carcinoma of the liver	2
atypical teratoid/rhabdoid tumor	1
epithelioid hemangioendothelioma	1
head & neck squamous cell carcinoma	1
hepatocellular carcinoma	1
leiomyosarcoma	1
myoepithelial carcinoma	1
myofibromatosis	1
prostate adenocarcinoma	1
synovial sarcoma	1
uterine carcinosarcoma	1
uterine corpus endometrioid carcinoma	1

Breakdown of diseases for RNA-Seq samples used in the pan-disease analysis.

After a discussion of available treatment options, the patient’s parents elected to start pazopanib. After three months, a chest CT scan showed progressive pulmonary metastases, and pazopanib was discontinued. After further discussion of treatment options and parental consent, the liquid formulation of ribociclib was obtained by a single-patient investigational new drug application and was started two months after pazopanib discontinuation. Twelve 28-day cycles of ribociclib were administered at the recommended pediatric dose (350 mg/m^2^ daily)^30^. CT scans showed lung nodule stability throughout treatment (Fig. 1c, Panels A and B). Median sternotomy and resection of 6 bilateral pulmonary nodules yielded 4 containing adequately excised myoepithelial carcinoma with variable hyalinization and fibrosis that may represent treatment response (5–30%). Postoperatively, 12 additional 28-day cycles of ribociclib were administered due to the perceived high risk of metastatic disease progression and the absence of significant ribociclib toxicity. CT chest imaging, most recently 2.5 years after completion of postoperative ribociclib, shows no evidence of myoepithelial carcinoma recurrence (Fig. 1c, Panel C). The patient is currently nine years old with no apparent long-term toxicity from ribociclib.

## Discussion

As the limits of cytotoxic chemotherapy have been reached and research has uncovered the genetic heterogeneity of cancer, oncologists have increasingly turned to genomic analysis to identify targeted therapies for those with rare and difficult-to-treat cancers. While there have been successes with targeted therapies in pediatric cancers, such as GD2-directed therapies in neuroblastoma^31^, entrectinib, larotrectinib^8^ and other TRK-fusion kinase inhibitors in NTRK-fusion-positive solid tumors^32^, BRAF, MEK, and pan-RAF inhibitors in gliomas with *BRAF* alterations^33^, a large proportion of pediatric solid tumors do not contain immediately targetable abnormalities^34^. To enhance the detection of druggable targets in pediatric malignancies, RNA-Seq is now being integrated into precision medicine workflows to identify gene fusions, but also increasingly to identify highly expressed oncogenes and oncogenic pathways that could serve as therapeutic targets^11,12,24^. However, gene expression information is often not prioritized due to 1) the lack of standard workflows impacting reproducibility and 2) insufficient preclinical and clinical evidence supporting using gene expression outliers as therapeutic targets.

Given the potential benefit of detecting abnormally expressed genes that may be therapeutically targeted^12,19,21,24,35–37^, we must standardize and validate gene expression outlier analysis of RNA-Seq data for pediatric tumors. Our case illustrates that the clinical utility of RNA-Seq for identifying therapeutic targets is highly dependent on an unbiased approach using large comparator datasets that have undergone uniform bioinformatic processing. This approach permits the identification of molecularly similar tumors that may not be expected based on tumor histology.

For cancers like myoepithelial carcinoma that are so rare that basic biologic research is difficult and clinical trials impossible, comparative RNA-Seq studies may be an important tool for both studying tumor biology and identifying beneficial therapies. *CCND2* overexpression has not been described previously in myoepithelial carcinoma and we found only one reference to immunohistochemical expression of *CDK4* in this disease^31^. Thus, in addition to identifying a targeted therapy that would never have been considered in this clinical setting, the gene expression analysis of our patient’s tumor contributed to our understanding of the biology of myoepithelial carcinoma and identified a potentially useful immunohistochemical marker for identifying future patients who might benefit from CDK4/6 inhibitors.

Further studies are needed to conclusively establish the scientific and clinical benefits of this framework, which compares individual tumors to a large comprehensive cohort of cancer genomic data to identify oncogenic pathway overexpression and associated therapeutic targets. However, our preliminary findings suggest that studying each tumor’s gene expression in the context of a growing repository of carefully analyzed tumors may have important implications for both scientific progress and patient care.

## Methods

### Consent

This patient was enrolled in the CARE IMPACT study^25^ conducted by investigators from the University of California Santa Cruz (UCSC) Treehouse Childhood Cancer Initiative and the Stanford University School of Medicine. Prior to any study procedures, informed consent (from patients over 18 years of age or the patient’s legal guardian for those under 18 years of age) and assent (from patients 7 to 18 years of age) were obtained according to institutional guidelines. Before initiation, this study was approved by the Institutional Review Boards at Stanford University (Human Subject Research Protocol 44179) and the University of California Santa Cruz (HS-FY2024-72). All handling of patient data was performed in accordance with the Declaration of Helsinki.

### DNA mutation analysis

The patient’s metastatic lung nodule was sent for DNA mutation testing at Foundation Medicine (https://www.foundationmedicine.com/portfolio, FoundationOne CDx panel).

### RNA sequencing protocol

The patient’s metastatic lung nodule was sent to Covance by Labcorp (Covance) for RNA-Seq. RNA was extracted with the Qiagen RNEasy kit. A sequencing library was prepared with the Illumina TruSeq Stranded mRNA Library Preparation and sequenced on an Illumina HiSeq 2500 sequencer to obtain 40–50 million reads.

### Patient data transfer

De-identified clinical and mutation information were extracted from the patient’s medical record and sent to the UC Santa Cruz (UCSC) Treehouse Childhood Cancer Initiative for analysis. UCSC Treehouse researchers never received direct patient identifiers. Secondary Treehouse identifiers (TH34_XXXX_S0X) were generated that could not be linked to direct patient identifiers. De-identified clinical data retrieved from the patient’s medical record included age, sex, race, ethnicity, cancer diagnosis, disease features, and treatment history.

The patient’s de-identified raw RNA-Seq dataset was obtained by UCSC from Covance. Covance uploaded the patient FASTQ files to UCSC Treehouse’s encrypted Amazon Web Services (AWS) bucket and provided quality metrics. The RNA-Seq file was downloaded from AWS to UCSC Treehouse’s secure servers for analysis. The RNA-Seq file and associated clinical metadata were managed using REDCap^38^ electronic data capture tools hosted by Treehouse.

### RNA-Seq sample quality control metrics

Our quality control (QC) framework^39^ was used to ensure sufficient quality of the RNA-Seq data for identifying overexpressed oncogenes and pathways. This method relies on counting MEND reads (Mapped to human genome, Exonic, and Non-Duplicate). Filtering the total pool of reads in an RNA-Seq sample for MEND reads results in a subpopulation of reads that reflect the integrity and quantity of RNA in the sample and indicate whether the data can be used for robust gene expression quantification.

### Sequencing data analysis and CARE computation pipelines

The patient’s RNA-Seq analysis (https://github.com/UCSC-Treehouse/pipelines) was uniformly performed as described previously^24^, with the following modifications. The most recent docker for the UCSC Treehouse RNA-Seq analysis pipeline was used (docker command: docker pull quay.io/ucsc_cgl/rnaseq-cgl-pipeline:3.3.4-1.12.3)^40^. For this case, the geneBody_coverage.py tool was not run.

The CARE pipeline was employed (https://github.com/UCSC-Treehouse/CARE) to identify clinically relevant oncogenes and oncogenic pathways in the patient’s tumor. Clinically relevant genes were designated as genes whose products could be directly or indirectly targeted through the downstream signaling pathway by an approved drug or an investigational agent in any phase of clinical development. The CARE pipeline and algorithm compare an RNA-Seq dataset from a focus sample to comparator cohorts and yield two outputs: (1) datasets molecularly similar to the focus sample and (2) genes that are abnormally expressed in the focus sample. The patient’s tumor RNA-Seq profile was compared to the publicly available v8 Treehouse polyA compendium (https://treehousegenomics.soe.ucsc.edu/public-data/). Tumors are considered molecularly similar if the Spearman correlation between their expression profiles is above the 95th percentile of all pairwise correlations within the compendium. Abnormally expressed genes are those exceeding the outlier threshold for the comparator cohort. Outlier thresholds are defined using the Tukey outlier detection method ((Interquartile Range)(1.5) + 75% Quartile). Pan-cancer and personalized pan-disease outlier analyses were performed. Pan-cancer outliers are those exceeding the outlier threshold defined by the entire v8 Treehouse polyA compendium (11,427 tumor RNA-Seq profiles from both adult and pediatric patients). Pan-disease outliers are genes with expression exceeding the outlier threshold from at least two of the four personalized pan-disease cohorts: (1) datasets from tumors with the same diagnosis as the focus sample, (2) molecularly similar RNA-Seq datasets (first degree neighbors), (3) first and second degree neighbors (first degree neighbors plus RNA-Seq datasets molecularly similar to them), and (4) datasets from diseases present among the top 6 most correlated datasets. Pan-cancer and pan-disease outliers were analyzed for enrichment of downstream pathways and signaling networks containing genes that could be targeted by available therapies.

For the pan-disease analysis, 58,581 genes from GENCODE Human Release 23 were used. For pan-cancer analysis an expression- and variance-filtered set of GENCODE 23 genes was used. First, the expression filter drops any gene where 80% or more of the samples have an expression of 0. Second, the variance filter sorts the remaining non-dropped genes and sorts them by the variance of their expression level across the cohort. 20% of these genes with the lowest variance are dropped regardless of absolute variance.

### Addressing batch effects in comparator cohorts

The v8 Treehouse polyA compendium was used in this manuscript. We release new versions of our compendia as we acquire new RNA-Seq datasets. Due to the reduction in biological signal that can accompany batch effect removal (https://academic.oup.com/gigascience/article/9/11/giaa117/5952607), we used two levels of review to detect potential batch effects. To detect batch effects at the group level, we reviewed a layout of RNA-Seq samples in the compendia based on gene expression similarity and annotated by disease^20^. Instances in which groups of RNA-Seq datasets with the same disease annotations are not adjacent in expression space are reviewed for likely errors. Secondly, at the time of analysis, we review a table of the samples most similar to the patient’s RNA-Seq profile and investigate any with disease or mutation annotations that would not be expected. In this way, we have identified both mistaken annotations and interesting biology (https://molecularcasestudies.cshlp.org/content/5/5/a004317.long).

### Analysis of overexpressed genes

The pan-cancer and pan-disease outlier gene lists from the patient’s CARE analysis were analyzed for enrichment of pathways and signaling networks containing genes that could be targeted by available therapies.

We used the Drug Gene Interaction Database (DGIdb)^41^ to identify which overexpressed genes could be targeted by clinically available inhibitors. DGIdb is an open-source project that searches through publications and other curated databases for known or potential interactions between human genes and available inhibitors. To focus our findings on drug targets with known cancer relevance, we set DGIdb to query drug-gene interactions in the following four curated databases: CIViC, Cancer Commons, My Cancer Genome, and My Cancer Genome Clinical Trial. DGIdb does not contain all known drug-gene interactions, nor does it guarantee that any interaction is an appropriate therapeutic intervention. To address these limitations, we conducted additional literature review and consulted published clinical cancer genomic studies. We prioritized studies that considered gene expression information when assessing the druggability of each gene.

We used the Molecular Signature Database (MSigDB)^42^ to identify significantly overexpressed cancer pathways in the patient’s tumor RNA-Seq dataset by conducting gene set overlap analysis, which computes statistically significant pathways between the input gene list of overexpressed genes and the gene sets in the chosen MSigDB collections “Hallmark Gene Sets” and “Canonical Pathways”.

### Immunohistochemistry staining

4 micrometer thick sections prepared from formalin fixed paraffin embedded (FFPE) tissue sampled from the metastatic lung nodule were sent to ARUP Laboratories for immunohistochemical straining for CDK4 (https://ltd.aruplab.com/Tests/Pub/2005534). For detection of Retinoblastoma (Rb) protein a purified mouse anti-human Rb protein antibody was used (BD Pharmingen, clone G3-245, catalog number 554136) on Leica Bond using 1:100 dilution and ER2 antigen retrieval protocol. The Rb assay was completed at Stanford clinical labs.

### Clinical genomics tumor board meeting

Upon completion of the CARE analysis, a clinical genomics tumor board was attended by the treating oncologist, additional pediatric oncologists, genomics scientists, bioinformaticians, data analysts, nurse practitioners, a genetic counselor, and various trainees. Prior to the session, clinicians were asked to avoid using HIPAA-protected patient identifiers during case discussions to protect patient privacy. The treating physician presented the patient’s history, including past treatment, current medical status, goals of care, and potential therapies being considered. A Treehouse data analyst presented the RNA-Seq data, including specimen quality metrics, gene expression findings, targeted agents identified, and literature supporting or refuting the use of the targeted agent in the patient’s tumor or similar tumors. DNA mutation panel results were also presented. Discussion focused on the strength of the analytical findings, the clinical evidence available to support the use of each identified treatment, and how to prioritize each option in the context of other available treatment options.

## Data Availability

The datasets generated and analyzed during the current study are available in our public data repository, https://treehousegenomics.soe.ucsc.edu/public-data/.

## Code availability

The code for the CARE algorithm used in this manuscript is available via this link https://github.com/UCSC-Treehouse/CARE. The underlying code for data analysis done in this study can be accessed via this link https://github.com/UCSC-Treehouse/CARE_IMPACT_analysis_for_manuscript. Calculations were performed and figures generated with R and RStudio using the following packages: tidyverse^43^, colorspace^44^, cowplot^45^, ggVennDiagram^46^, ggforce^47^, ggrepel^48^, gridExtra^49^, haven^50^, janitor^51^, jsonlite^52^, kableExtra^53^, khroma^54^, knitr^55^, networkD3^56^, RColorBrewer^57^, redcapAPI^58^, UpSetR^59^, webshot^60^.
